# Jasmonic Acid and Salicylic Acid in Regulating Plant Cadmium Accumulation and Tolerance: Mechanisms and Crosstalk

**DOI:** 10.3390/plants15162546

**Published:** 2026-08-21

**Authors:** Tianyu Gu, Shilong Zhao, Xiaoyi Zhang, Siying Chen, Yan Gao, Jiashi Peng

**Affiliations:** 1 Hunan Key Laboratory of Economic Crops Genetic Improvement and Integrated Utilization, Hunan University of Science and Technology, Xiangtan 411201, China; zhaoshilong@mail.hnust.edu.cn (S.Z.); xiaoyizhang2026@163.com (X.Z.); swing9510c@163.com (S.C.); blessedgy@hnust.edu.cn (Y.G.); 2 Key Laboratory of Ecological Remediation and Safe Utilization of Heavy Metal-Polluted Soils, College of Life and Health Sciences, Hunan University of Science and Technology, Xiangtan 411201, China

**Keywords:** jasmonic acid, salicylic acid, cadmium stress, phytohormone crosstalk, transporter regulation, phytoremediation

## Abstract

Cadmium (Cd) is a highly toxic non-essential heavy metal that severely impairs plant growth, compromises crop yield and quality, and threatens food safety and human health. Jasmonic acid (JA) and salicylic acid (SA) are well-characterized endogenous phytohormones that serve as central regulators in modulating plant adaptive responses to Cd stress. This review comprehensively synthesizes current knowledge on the involvement of JA and SA in regulating Cd accumulation and tolerance in plants, including their Cd-induced biosynthetic dynamics and signaling transduction pathways, as well as their functions in restricting Cd uptake and translocation, modulating chelation and sequestration, reinforcing antioxidant defense systems and protecting photosynthetic apparatus. Moreover, we analyze the antagonistic and synergistic crosstalk between JA and SA, and discuss how this interplay shapes Cd resilience in plants. Finally, the application potential of JA and SA in developing Cd-safe crops and phytoremediation in Cd-contaminated farmland is explored. This review provides a systematic analysis of the regulatory roles of JA and SA in plant responses to Cd stress, along with an in-depth discussion of their crosstalk. These insights contribute to the rational development of hormone-based breeding strategies for Cd-safe crops and the optimization of agronomic management practices.

## 1. Introduction

Cadmium (Cd) contamination in agricultural soils has emerged as a critical environmental challenge that constrains global crop productivity and compromises food security [1,2,3]. As a highly bioavailable and non-essential heavy metal, Cd induces oxidative stress, impairs photosynthetic efficiency, disrupts nutrient uptake, and causes cellular damage in plants even at trace concentrations [4]. Meanwhile, Cd accumulates in the edible tissues of crops and threatens human health along the food chain [5,6]. To cope with Cd toxicity, plants have developed a suite of coordinated defense mechanisms, including restriction of root uptake, vacuolar compartmentalization, cell wall immobilization, chelation by thiol-rich ligands (e.g., phytochelatins and metallothioneins), and activation of antioxidant enzymatic systems [7,8,9].

Phytohormones have been extensively investigated as central coordinators of plant adaptive responses to abiotic stresses, including heavy metal toxicity [10,11]. Among the canonical phytohormone classes, jasmonic acid (JA) and salicylic acid (SA) have attracted growing attention for their distinct yet functionally interconnected roles in modulating Cd tolerance [12,13,14]. JA, an oxylipin-derived signaling molecule synthesized via the octadecanoid pathway, and SA, a phenolic compound predominantly produced through the isochorismate pathway, were initially characterized for their functions in pathogen and herbivore defense [15,16]. Accumulating evidence indicates that Cd stress rapidly elevates endogenous JA and SA levels, and exogenous application of either hormone markedly reduces Cd accumulation and mitigates Cd-triggered damage in a wide range of plant species. Nevertheless, the molecular mechanisms through which JA and SA regulate plant Cd tolerance, as well as the crosstalk between the two signaling pathways under Cd stress, remain to be thoroughly elucidated.

To systematically synthesize the current knowledge on JA- and SA-regulated Cd accumulation and tolerance in plants, especially the crosstalk between the two signaling pathways, this review focuses on: (i) the dynamics of JA and SA biosynthesis and signal transduction under Cd stress; (ii) their individual molecular contributions to Cd detoxification and homeostasis; (iii) the crosstalk between JA and SA pathways under Cd stress; and (iv) the application of JA and SA in crop improvement and phytoremediation to cope with Cd pollution. This work aims to provide theoretical references for hormone-mediated crop improvement and phytoremediation applications.

## 2. Jasmonic Acid in Cadmium Tolerance

### 2.1. JA Biosynthesis and Signaling Under Cd Stress

α-Linolenic acid is the primary precursor for JA biosynthesis. Firstly, α-Linolenic acid is released from chloroplast membranes, and enters the octadecanoid pathway [17,18,19]. This substrate then undergoes sequential enzymatic conversion catalyzed by lipoxygenase (LOX), allene oxide synthase (AOS), and allene oxide cyclase (AOC), yielding 12-oxo-phytodienoic acid (OPDA) [20,21]. OPDA is subsequently metabolized to produce bioactive jasmonic acid (JA) and its physiologically active conjugate, jasmonoyl-isoleucine (JA-Ile) [22]. Methyl jasmonate (MeJA), another bioactive derivative of JA, originates from the methyltransferase-catalyzed methylation of JA [23]. Characterized by volatility, MeJA acts as an endogenous mobile signal translocating over long distances to trigger plant defensive responses [24]. Under cadmium (Cd) stress, endogenous JA levels increase markedly in plants due to transcriptional upregulation of JA biosynthetic genes. In rice (*Oryza sativa*), Cd treatment markedly elevated root JA levels, accompanied by significant upregulation of JA biosynthetic genes (*OsLOX*, *OsAOS*, and *OsAOC*) and JA-responsive transcription factors [25,26]. Cd exposure also activates the JA synthetic genes and increases the accumulation of endogenous JA in *Arabidopsis* and potato (*Solanum tuberosum*) [27,28].

**Figure 1:**
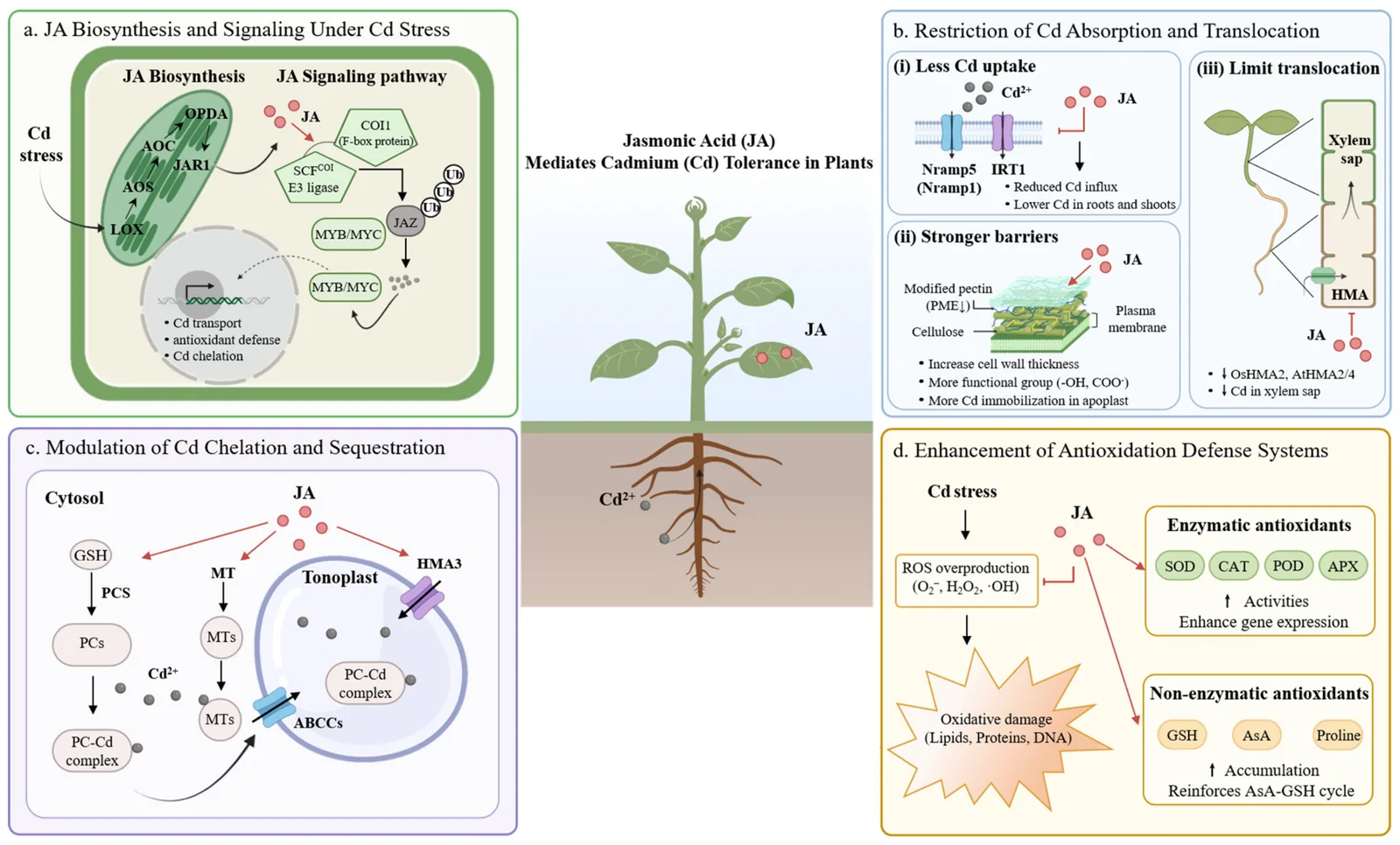
The regulatory mechanism of JA in plant Cd tolerance. (**a**) JA Biosynthesis and Signaling: Cd stress induces JA biosynthesis. Bioactive JA triggers degradation of JAZ repressors via the SCF-COI1 complex, releasing transcription factors (e.g., MYB, MYC) to activate downstream defense genes. (**b**) Restriction of Cd Absorption and Translocation: JA limits Cd accumulation by downregulating uptake transporters (e.g., Nramp5, IRT1) and reinforcing cellular barriers to immobilize Cd. It also restricts root-to-shoot translocation by inhibiting xylem loading. (**c**) Modulation of Chelation and Vacuolar Sequestration: JA upregulates PCs and MTs synthesis to chelate cytosolic Cd^2+^ and promotes vacuolar sequestration via transporters (e.g., ABCCs, HMA3). (**d**) Activation of Antioxidant Defense: JA induces enzymatic (e.g., SOD, CAT, APX) and non-enzymatic (e.g., GSH, AsA, Proline) antioxidants to scavenge excessive ROS, mitigating oxidative damage and maintaining redox homeostasis. ↑ in (**d**) means increase. Arrows and bars indicate promotion and inhibition, respectively.

JA signaling is transduced via the F-box protein COI1 (Coronatine Insensitive 1), a core component of the SCF (Skp1-Cullin-F-box protein complex) -COI1 ubiquitin ligase complex. Upon perception of JA-Ile, this complex mediates proteasomal degradation of JAZ (Jasmonate ZIM-domain) repressor proteins [29,30,31]. JAZ degradation releases downstream transcription factors, including various MYC and MYB (Myeloblastosis viral oncogene homolog and Myelocytomatosis viral oncogene homolog) family members, which subsequently activate transcriptional networks governing Cd transport, antioxidant defense, and Cd chelation [26,32,33]. Consistently, rice mutants defective in JA biosynthesis (e.g., *OsAOS*) or perception (e.g., *OsCOI1*) exhibit heightened Cd sensitivity and increased Cd accumulation [26]. In tomato (*Solanum lycopersicum*), SlMYC2 directly upregulates root-localized flavonoid synthase SlF3′H and boosts flavonoid production, which in turn inhibits the root-to-shoot long-distance translocation of Cd [33]. Therefore, JA serves as an indispensable signaling hub for initiating rapid stress responses and establishing adaptive tolerance to Cd toxicity in plants (Figure 1a).

### 2.2. Restriction of Cd Absorption and Translocation

JA has been reported to reduce Cd accumulation across multiple plant species. Modulation of rhizospheric Cd uptake constitutes the first barrier of plant defense against Cd phytotoxicity, and JA exerts potent regulatory effects on this process. In rice, MeJA application depressed the expression of cation transporter genes (*OsNramp1* and *OsNramp5*, *Natural Resistance-Associated Macrophage Protein*), resulting in reduced Cd influx and decreased Cd accumulation in roots and shoots by 10.15% and 36.39%, respectively [34]. Correspondingly, the *OsAOS-* or *OsCOI1*-RNAi plants accumulated significantly more Cd than wild-type plants, accompanied by elevated transcript levels of *OsNramp5* and *OsIRT1* (*Zrt-/Irt-like Protein*), confirming that the JA module directly represses Cd transporter expression under Cd stress [26]. Exogenous MeJA also restricts root Cd uptake in tomato, *Solanum nigrum*, *Arabidopsis*, wheat (*Triticum aestivum*), and pigeon pea (*Cajanus cajan*) [28,35,36,37,38,39].

Beyond limiting root Cd uptake, JA also impedes the radial translocation of Cd toward the xylem. This is partly achieved through JA-mediated modulation of cell wall composition, which enhances Cd immobilization in the root apoplast. In rice, MeJA treatment increased Cd contents in the cell wall fraction, coinciding with enhanced cell wall thickness and altered cell wall polysaccharide (pectin, cellulose, hemicellulose) content [40,41,42]. Fourier-transform infrared (FTIR) spectroscopy analysis implied that functional groups such as hydroxyl (-OH) and carboxylate (COO^−^) on cell wall polymers contribute to Cd binding [34]. Comparable effects have been observed in *Cosmos bipinnatus* [43,44], tomato [37,45] and apple rootstocks [32], where optimal MeJA dosing reduces symplastic Cd entry by modulating pectin methylesterase (PME) activity, remodeling pectin molecular conformation, and elevating both the contents and Cd-binding capacity of cell wall polysaccharides.

In addition, exogenous MeJA inhibits root-to-shoot Cd translocation. This is evidenced by suppressed expression of *HMA2* (a heavy metal-associated protein facilitating long-distance Cd transport) in rice and wheat roots and declined Cd levels in xylem sap [26,34,39]. In *Arabidopsis*, MeJA application decreased the expression of *AtHMA2* and *AtHMA4*, whereas mutation of the JA synthesis gene *AtAOS* resulted in marked upregulation of these transporters and heightened Cd sensitivity [28]. Collectively, JA orchestrates a coordinated regulation of Cd transporter expression and cell wall properties, thereby effectively reducing Cd translocation to aerial tissues, and protecting photosynthetic organs from Cd-induced toxicity (Figure 1b).

### 2.3. Modulation of Cd Chelation and Sequestration

Upon Cd influx into the cell, cytoplasmic phytochelatins (PCs) and metallothioneins (MTs) perform further chelation and immobilization of Cd [46,47]. PCs are synthesized from glutathione (GSH) by phytochelatin synthase PCS (PC synthase) in the cytosol, and bind Cd with high affinity, forming low-mobility PC–Cd complexes that are subsequently transported into vacuoles via the ATP-binding cassette transporters ABCC1 and ABCC2 [48,49]. MTs constitute another class of Cd-detoxifying compounds. They are cysteine-rich, low-molecular-weight metal-binding proteins widely distributed across plant and animal taxa [50]. Their primary role in Cd detoxification involves sequestration of free Cd^2+^ ions, and stabilization of cellular metal ion homeostasis [51,52].

In pak-choi (*Brassica chinensis*), exogenous JA treatment elevated levels of GSH, PCs and MTs in the roots, thereby effectively enhancing Cd retention and detoxification [53]. In parallel, JA confers enhanced Cd tolerance in rice, largely attributable to elevated PC accumulation that accelerates Cd chelation and subsequent vacuolar sequestration [25,41]. In addition, MeJA markedly induces the expression of MT-encoding genes in two mangrove species, *Kandelia obovate* [54] and *Avicennia marina* [55]. Such induction restricts the root-to-shoot translocation of Cd, diminishes foliar Cd accumulation, and ultimately enhances plant resistance against Cd stress.

JA further modulates the sequestration of Cd through the regulation of tonoplast-localized transporters and cell wall binding. In rice, JA promotes Cd retention in roots by upregulating the expression of genes involved in vacuolar sequestration, such as *OsHMA3* and *OsABCG36* [42]. Analogous mechanisms have also been reported in wheat [39,56] and pigeon pea [38]. Besides the vacuole, the cell wall is another important site for metal sequestration in plants [57,58,59,60]. Transcriptomic profiling in the Cd hyperaccumulator *Sedum alfredii* revealed that JA modulated the expression of numerous genes involved in cell wall modification, vacuolar transport, and metal chelation, facilitating Cd immobilization in protective compartments [61]. This coordinated regulation of chelation and sequestration ensures that Cd is effectively detoxified and compartmentalized away from sensitive metabolic sites (Figure 1c).

### 2.4. Enhancement of Antioxidant Defense Systems

Cd toxicity is primarily manifested through the excessive generation of reactive oxygen species (ROS), including superoxide anion (O_2_•^−^), hydrogen peroxide (H_2_O_2_), and hydroxyl radicals (•OH), which cause oxidative damage to lipids, proteins, and nucleic acids [62]. JA application has been shown to restore oxidative homeostasis by enhancing both enzymatic and non-enzymatic antioxidant systems [63,64].

In rice, spinach (*Spinacia oleracea*) [65], green amaranth (*Amaranthus viridis*) [66] and faba bean (*Vicia faba*) [67], exogenous JA application alleviated Cd-induced growth inhibition by reducing Cd uptake, inhibiting H_2_O_2_ accumulation, and enhancing activities of antioxidant enzymes like superoxide dismutase (SOD), catalase (CAT), peroxidase (POD), and ascorbate peroxidase (APX). Beyond enzymatic antioxidants, JA also promotes the accumulation of non-enzymatic antioxidants such as GSH and ascorbate (AsA), thereby maintaining cellular redox homeostasis in cucumber (*Cucumis sativus*) [68], mustard (*Brassica juncea*) [69], *K. obovate* [54], and *S. alfredii* [61]. Transcriptome analysis of potato under Cd stress identified a suite of JA-responsive antioxidant-related genes. Among them, StJAZ14 was identified as a negative regulator of JA signaling, and was downregulated upon Cd exposure, thereby derepressing antioxidant gene expression [27]. The physical interaction between StJAZ14 and StBZR1, a key component of the brassinosteroid (BR) signaling pathway, preliminarily implies potential hormonal crosstalk between JA and BR under Cd stress. These findings provide a theoretical basis for the subsequent crop improvement for increasing plant Cd tolerance via combined JA and BR treatments [27]. In soybean (*Glycine max*) roots, JA markedly elevated the activity and protein abundance of heme oxygenase 1 (HO-1). Application of HO-1 inhibitor ZnPPIX completely abolishes the protective effects conferred by JA, demonstrating that HO-1 acts as an essential downstream component of the JA-mediated antioxidant pathway [70,71].

In addition, exogenous co-treatment of JA with other bioactive compounds elicits synergistic enhancement of plant antioxidant capacity. In chickpea (*Cicer arietinum*) [64], combined JA and gibberellic acid (GA_3_) treatment mitigated Cd toxicity through comprehensive oxidative stress mitigation, significantly reducing malondialdehyde (MDA) levels and ROS accumulation while restoring the activities of SOD, CAT and POD. In wheat, two distinct combinatorial strategies, i.e., JA priming followed by spermidine application, and co-treatment with MeJA and the nitric oxide donor sodium nitroprusside, both effectively suppressed lipid peroxidation, hydrogen peroxide (H_2_O_2_) accumulation, and electrolyte leakage; concurrently enhanced antioxidant enzyme activities; and ultimately relieved Cd-induced growth retardation [72,73].

Moreover, joint application of JA with essential mineral nutrients similarly improves plant resilience to Cd stress via modulation of antioxidant metabolism. In pea (*Pisum sativum*), combined treatment with JA and potassium (K) efficiently recovered growth upon Cd exposure and increased the activities of nitrate reductase (NRA), nitrite reductase (NiRA) and antioxidant enzymes [74]. In *Mentha arvensis*, co-application of MeJA and nitrogen (N) robustly activated the ROS-scavenging enzymatic system, thereby counteracting Cd-triggered ROS overproduction, and reversing Cd-induced stomatal closure [75]. Likewise, synergistic treatment with MeJA and selenium (Se) substantially alleviates photosynthetic depression in hot pepper (*Capsicum annuum*) under Cd stress, and lowers foliar Cd accumulation by elevating antioxidant enzyme activities [76].

JA-dependent activation of antioxidant defenses under Cd exposure has been reported across diverse plant species, including tobacco (*Nicotiana tabacum*) [77], water spinach (*Ipomoea aquatica*) [78], okra (*Abelmoschus esculentus*) [79], *S. nigrum* [35], pigeon pea [38] and *A. marina* [55]. These findings establish JA-mediated antioxidant regulation as a conserved and pivotal adaptive mechanism enabling plants to mitigate Cd-induced oxidative damage (Figure 1d).

## 3. Salicylic Acid in Cadmium Tolerance

### 3.1. SA Biosynthesis, Signaling, and Dual Roles in Cd Responses

SA is synthesized in plants via two major pathways: the isochorismate synthase (ICS) pathway in chloroplasts and the phenylalanine ammonia-lyase (PAL) pathway in the cytosol [80,81,82,83]. In rice and *Arabidopsis*, Cd stress induces substantial accumulation of endogenous SA in roots, creating a hormone signal that orchestrates downstream defensive responses [13,84]. In wheat, Cd exposure induced SA synthesis predominantly in leaves via the PAL pathway, and Cd tolerance was positively correlated with the extent of SA induction [85].

SA perception is mediated by multiple receptor proteins, among which NPR1 (Non-expressor of Pathogenesis-Related genes 1) serves as a master transcriptional regulator of SA-responsive gene expression [86]. Upon SA binding, NPR1 undergoes redox-dependent conformational changes, facilitating its nuclear translocation and subsequent interaction with TGACG motif-binding factors (TGA) to activate expression of antioxidant and detoxification genes [87]. Consistent with this, the *Arabidopsis npr1* mutant exhibits heightened Cd sensitivity and impaired Cd-induced SA accumulation upon Cd exposure [88], confirming NPR1’s indispensable role in SA-mediated Cd tolerance.

**Figure 2:**
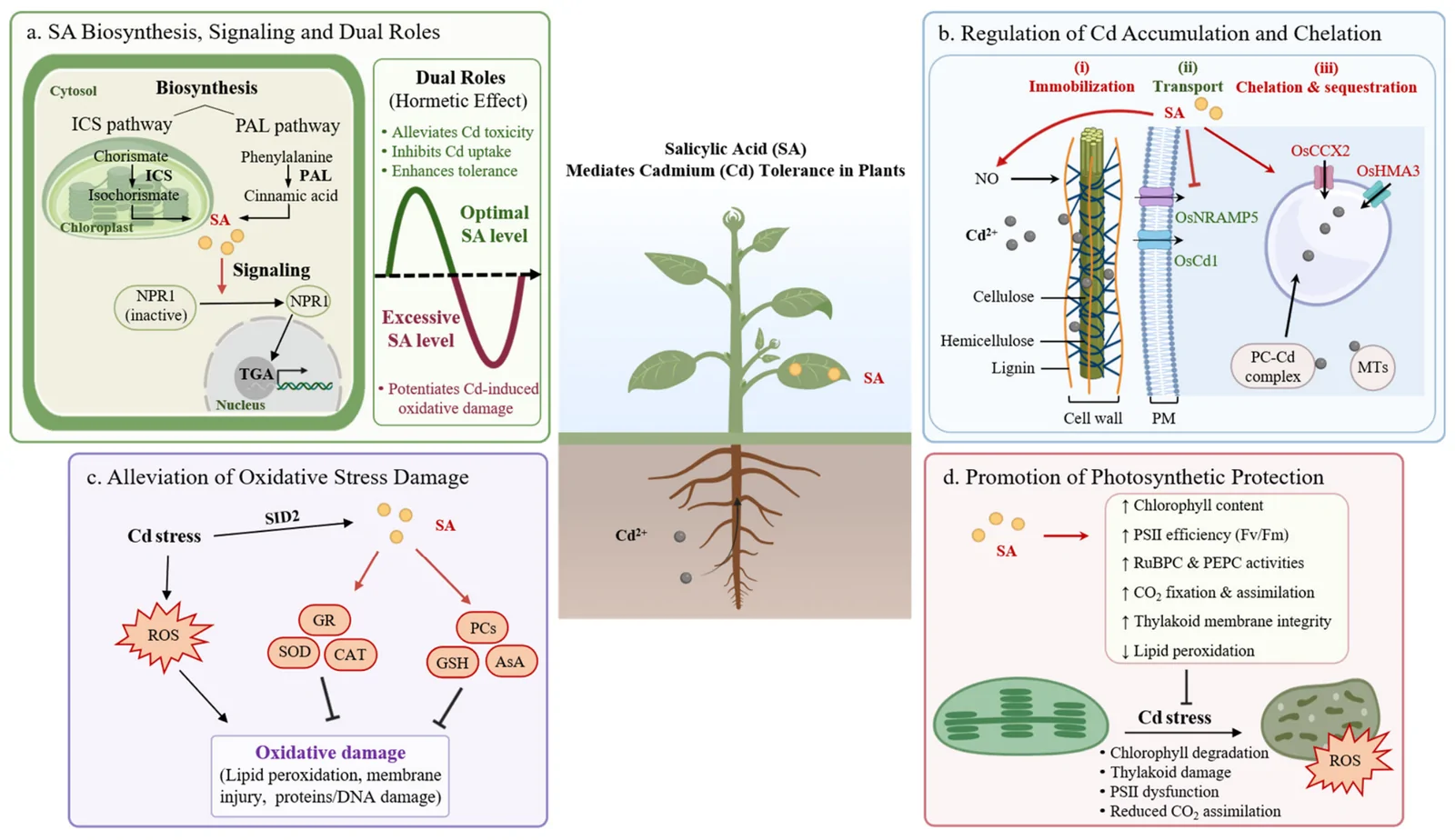
The regulatory mechanism of SA in plant Cd tolerance. (**a**) SA Biosynthesis, Signaling, and Dual roles: SA is synthesized via the ICS and PAL pathways; downstream signaling involves the activation of TGA transcription factors by NPR1. SA exhibits a hormetic effect where optimal levels alleviate Cd toxicity, while excessive levels may potentiate oxidative damage. (**b**) Regulation of Cd Accumulation and Chelation: SA mitigates Cd toxicity by promoting cell wall immobilization (via NO-mediated lignin and cellulose enhancement), inhibiting uptake through transporters (e.g., OsNRAMP5, OsCd1), and enhancing chelation (via PCs and MTs) and vacuolar sequestration (via OsHMA3 and OsCCX2). (**c**) Alleviation of Oxidative Stress Damage: SA enhances the antioxidant defense system (e.g., SOD, CAT, GR, GSH, AsA) to scavenge ROS and protect against lipid peroxidation and DNA damage. (**d**) Promotion of Photosynthetic Protection: SA maintains chlorophyll content and PSII efficiency, enhances CO_2_ fixation, and preserves thylakoid membrane integrity under Cd stress. ↑ and ↓ in (**d**) means increase and decrease. Arrows and bars indicate promotion and inhibition, respectively.

Notably, SA exhibits concentration-dependent dual effects in Cd stress responses. At appropriate concentrations, exogenous SA alleviates Cd toxicity by promoting growth maintenance and reducing Cd uptake, whereas excessive SA exerts phytotoxic effects of Cd. This phenomenon has been observed in multiple plant species subjected to SA treatment, including barley (*Hordeum vulgare*) [89,90], wheat [91,92], flax (*Linum usitatissimum*) [93,94] and rice [95]. The *Arabidopsis* SA-reducing transgenic line overexpressing *NahG* (naphthalene hydroxylase G, which degrade endogenous SA) showed enhanced tolerance to Cd compared with wild-type plants, whereas the SA over-accumulating mutant *snc1* (suppressor of *npr1-1*, constitutive 1) exhibited increased Cd sensitivity [88]. These findings underscore the importance of SA homeostasis for Cd tolerance and suggest a hormetic dose–response relationship between SA and Cd tolerance rather than a simple linear association. An optimal concentration of SA could function as a signaling molecule that activates antioxidant defense systems. In contrast, elevated SA concentrations can induce metabolic disorder and oxidative damage, thereby exerting antagonistic effects on Cd tolerance [96]. Elucidating the molecular mechanisms underlying this concentration-dependent modulation of SA in Cd stress responses remains an important direction for future research (Figure 2a).

### 3.2. Regulation of Cd Accumulation and Chelation

SA modulates Cd accumulation and tolerance through multiple physiological regulations, including root uptake, translocation inside the plant, and metal chelation [13]. In rice, SA treatment modulated Cd transport-related gene expression: downregulating *OsNRAMP5* and *OsCd1* for Cd uptake suppression, and upregulating *OsHMA3* and *OsCCX2* for Cd vacuolar sequestration [95,97,98,99,100]. Similarly, exogenous SA reduces Cd contents in the shoots of lettuce (*Lactuca sativa*) [101] and spinach [102] by regulating transporters (e.g., NRAMP and HMA family) associated with Cd uptake and translocation. By contrast, hyperaccumulators such as *S. alfredii* respond to SA in the opposite manner. SA upregulates Cd transport and detoxification-related genes to facilitate Cd absorption and accumulation [103,104].

Stimulating the biosynthesis of PCs and MTs constitutes another mechanism underlying SA-mediated Cd detoxification in plants. SA-induced elevation of PCs levels promotes the formation of stable, low-mobility PC-Cd complexes that are actively transported into vacuoles via ABCC-type transporters, effectively lowering bioavailable cytosolic Cd^2+^ [13,105]. Likewise, SA enhances MT synthesis in pak-choi, contributing to intracellular Cd detoxification via high-affinity chelation [53].

Moreover, SA induces cell wall structural modifications that increase the capacity of the root cell wall to sequester Cd outside the living protoplast. Cd binds with high affinity to carboxyl and hydroxyl groups of cell wall components, including pectin, lignin, and hemicellulose, and an expanded or chemically modified cell wall provides additional Cd-binding capacity [106,107]. In rice, SA promotes pectin synthesis and demethylation as well as lignin biosynthesis via nitric oxide signaling pathways, resulting in increased Cd retention in root cell walls [95,108,109]. In *Fagopyrum tataricum*, exogenous SA enhances root Cd retention by promoting root cell wall biosynthesis genes and increasing the allocation of Cd to the cell wall [110]. Comparable mechanisms as described above have been extensively documented in diverse plant species, including tomato [111] and willow (*Salix suchowensis*) [112] (Figure 2b).

### 3.3. Alleviation of Oxidative Stress Damage

SA also functions as a pivotal regulator of redox homeostasis in plants under Cd stress. In rice roots, SA application mitigated Cd-induced oxidative stress by modulating the expression of antioxidant enzyme genes and maintaining the activities of SOD, CAT, and GR, thereby reducing lipid peroxidation and preserving membrane integrity [97,113,114]. Pretreatment with SA significantly alleviates Cd-induced damage to plant growth, membrane stability and photosynthetic pigments in wheat, and this protection is primarily attributable to enhanced antioxidant enzyme activity [92,115]. Similar results have also been reported in other plant species, such as barley [90], potato [116], flax [117], melon (*Cucumis melo*) [118], mustard [119], *Chenopodium quinoa* [120] and *Pterocarya fraxinifolia* [121]. Furthermore, SA-deficient *NahG*-overexpressing Arabidopsis exhibits markedly heightened sensitivity to Cd toxicity, accompanied by severe photochemical impairment, excessive ROS accumulation and autophagic cell death; these phenotypes are fully rescued by exogenous SA application [84].

In addition, SA promotes the accumulation of GSH and AsA. Elevated GSH levels under SA treatment not only directly scavenge ROS but also serve as the essential substrate for phytochelatin biosynthesis [8,122]. In *Arabidopsis*, Cd stress induced SA accumulation via transcriptional upregulation of *SID2* (isochorismate synthase). SA depletion in *sid2* mutants impairs the synthesis and recycling of GSH, as evidenced by repressed expression of serine acetyltransferase (SAT), decreased accumulation of non-protein thiols and PCs, and reduced transcript abundance and activity of glutathione reductase 1 (GR1), finally resulting in severe Cd hypersensitivity [14,123]. SA application in *Medicago sativa* induced *MsHO1* (Haem oxygenase-1) gene expression and protein accumulation, leading to activation of downstream antioxidant enzymes and restoration of GSH and AsA pools to re-establish redox homeostasis under Cd stress [124]. In bean plants [125,126,127], SA treatment significantly modulates AsA and GSH homeostasis under Cd exposure, further underscoring its role in fine-tuning the antioxidant response to Cd-triggered oxidative damage (Figure 2c).

### 3.4. Enhancement of Photosynthetic Protection

Photosynthesis is highly susceptible to Cd toxicity, which induces structural damage to chloroplasts, impairs chlorophyll synthesis, inhibits electron transport, and reduces carbon fixation and assimilation [128]. Accumulating evidence reveals that SA treatment mitigates Cd-induced photosynthetic dysfunction by preserving photosynthetic pigment integrity, enhancing chlorophyll accumulation, sustaining chloroplast photochemical activity and RuBisCO-mediated carboxylation capacity, thereby maintaining photosynthetic homeostasis under Cd stress [13].

Exogenous SA application has been consistently demonstrated to attenuate Cd-induced chlorophyll degradation across diverse plant species, including rice [113], tomato [129], spinach [102], green amaranth [66], milk thistle (*Silybum marianum*) [130], pea [131] and flax [132]. In maize (*Zea mays*), SA restores plant growth, increases photosynthetic CO_2_ fixation rate, enhances the activities of ribulose-1,5-bisphosphate carboxylase (RuBPC) and phosphoenolpyruvate carboxylase (PEPC), and elevates chlorophyll content under Cd stress, while it reduces lipid peroxidation, proline accumulation and electrolyte leakage [133,134]. In wheat, SA maintained the photosynthetic rate under Cd stress primarily by stabilizing the thylakoid membrane architecture and suppressing chloroplastic lipid peroxidation [91,92]. Furthermore, the SA-mediated protection of Photosystem II (PSII) was verified by improved chlorophyll fluorescence parameters (Fv/Fm) and reduced Cd accumulation in photosynthetic tissues.

In the same way, endogenous SA contributes to sustained photosynthetic efficiency under Cd stress in *Monochoria korsakowii* by reinforcing antioxidant defense systems, promoting chelator accumulation, and synergistically interacting with zeatin (ZT) and methyl salicylate (MeSA) to support carbon assimilation [135]. In *Arabidopsis* [88], genetic modification of basal SA levels altered plant responses to Cd stress, with SA levels positively correlating with photosynthetic performance and Cd tolerance (Figure 2d).

## 4. Crosstalk Between JA and SA Under Cadmium Stress

Both JA and SA alleviate Cd toxicity through convergent physiological mechanisms. Both phytohormones regulate the expression of Cd transporter genes to limit root uptake and translocation of Cd inside the plant and promote cell wall remodeling and metal-chelator biosynthesis to increase extracellular and intracellular chelation of Cd, alleviating ROS-induced oxidative damage via coordinated activation of antioxidant systems. SA has also been reported to protect the photosynthetic apparatus under Cd stress via chlorophyll stabilization, thylakoid-membrane maintenance and sustained PSII efficiency [12,13,14]. Although JA and SA converge on analogous downstream physiological outcomes in conferring Cd tolerance, their upstream signaling pathways exhibit marked divergence (Figure 1 and Figure 2). The precise molecular mechanisms by which each hormone orchestrates these shared physiological responses remain incompletely elucidated and warrant systematic investigation. Notably, JA and SA do not merely function independently under Cd stress, but also form an intricate antagonistic and synergistic crosstalk network to jointly regulate plant Cd accumulation and tolerance. The signaling crosstalk between the two hormones involves multiple levels of physiological metabolism, gene expression, and signal transduction with other phytohormones, which greatly improves the accuracy and flexibility of plant response to Cd stress.

### 4.1. Antagonistic Interactions and Signaling Balances

The crosstalk between JA and SA has been most extensively characterized in plant–pathogen interactions, establishing the “mutual antagonism” paradigm. Biotrophic pathogens predominantly induce SA-dependent defenses (systemic acquired resistance, SAR), while necrotrophic pathogens trigger JA-mediated responses (induced systemic resistance, ISR) [136]. The antagonistic relationship is mediated primarily through NPR1-dependent mechanisms. SA-induced NPR1 accumulation suppresses JA-responsive gene expression, and conversely, JA signaling can inhibit SA accumulation through the action of MYC2 on isochorismate synthase expression [137,138]. Whether similar antagonistic mechanisms operate under Cd stress remains to be investigated, and *npr1* and *myc2* mutants can be used as genetic materials to investigate whether NPR1- and MYC2-dependent regulators are involved in JA SA antagonistic interactions under Cd stress.

Under Cd stress, antagonistic interactions between JA and SA operate within a broader regulatory context. The SA-overaccumulating mutant *snc1* displayed altered JA responses and increased Cd sensitivity, suggesting that excessive SA may disrupt JA-mediated Cd defense mechanisms [88,139]. In the Cd-hyperaccumulator *Noccaea praecox*, high leaf Cd appeared to suppress biotic-stress-induced SA accumulation while triggering JA signaling. Cd exposure alone tended to increase leaf SA but not JA; however, either fungal attack or mechanical wounding decreased SA and induced JA in Cd-rich leaves [140]. These observations reveal a complex, context-dependent nature of JA–SA crosstalk during Cd stress.

**Figure 3:**
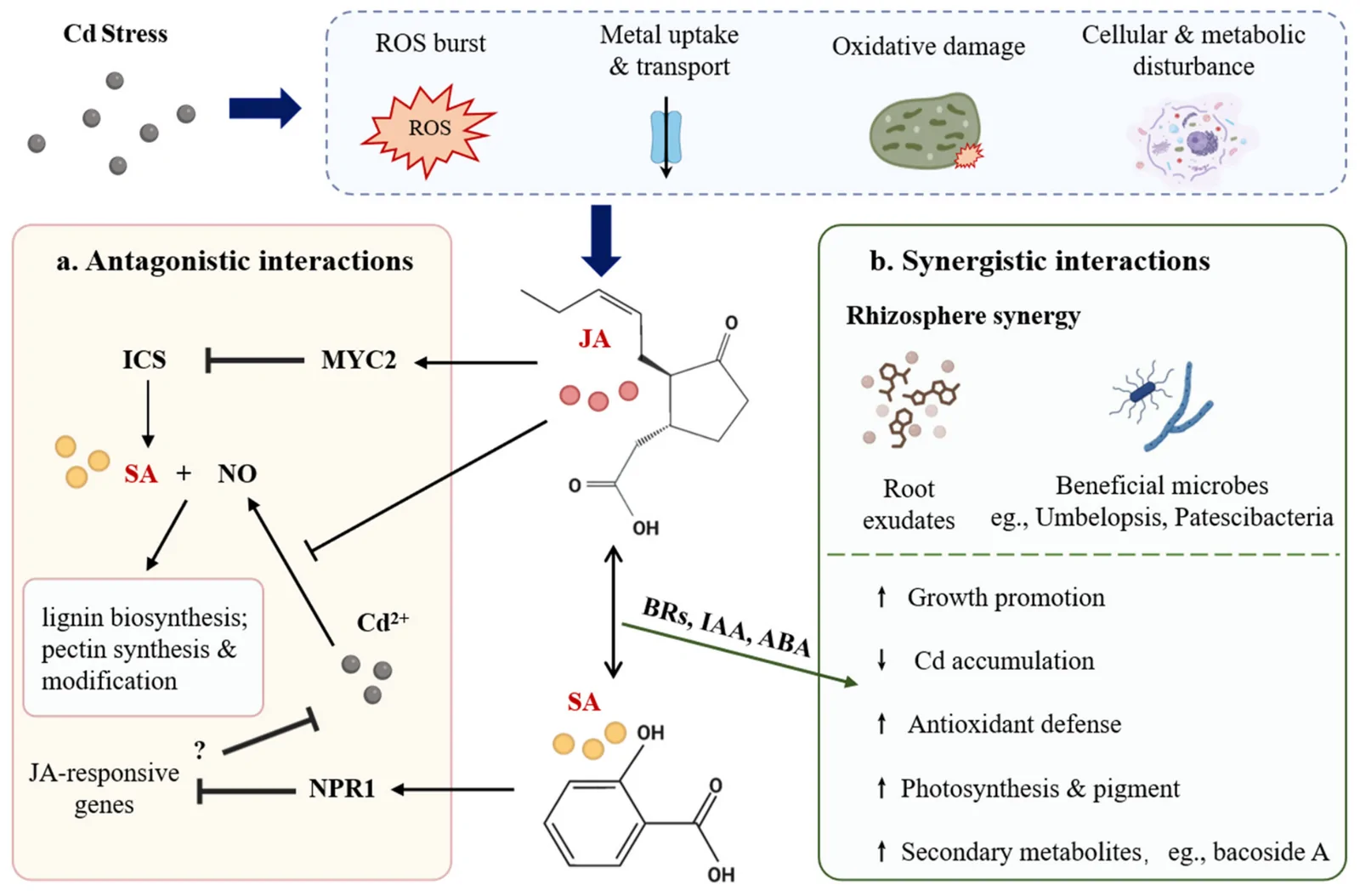
The Antagonistic and Synergistic Interactions of JA and SA Under Cd Stress. Plants experience rapid ROS bursts, excessive metal uptake, and cellular metabolic disturbances upon Cd exposure. The signaling interplay between JA and SA plays a critical role in orchestrating adaptive responses. (**a**) Antagonistic interactions: (i) JA alleviates Cd stress by suppressing Cd-induced NO production, whereas the co-regulation of SA and NO facilitates structural reinforcement via lignin and pectin modification to block Cd influx into cells. (ii) SA-induced NPR1 protein accumulation represses the expression of JA-responsive genes. Conversely, JA signaling inhibits ICS expression via MYC2 to reduce SA biosynthesis. Whether this regulatory pattern exists under Cd stress remains to be investigated. (**b**) Synergistic interactions: In the rhizosphere and at the systemic level, JA and SA can coordinate with beneficial microbiomes to enhance plant growth and Cd tolerance. This synergy is manifested through the: (i) promotion of plant growth; (ii) reduction in Cd accumulation; (iii) elevation of antioxidant defense; (iv) protection of photosynthesis and pigments; and (v) stimulation of specialized secondary metabolites (e.g., bacoside A) to mitigate toxicity. ↑ and ↓ in (**b**) means increase and decrease. Arrows and bars indicate promotion and inhibition, respectively.

This antagonistic balancing act extends to interactions with other signaling molecules, particularly nitric oxide (NO). Excessive NO caused by Cd stress can lead to nitrosative damage; yet exogenous JA markedly suppresses this NO overaccumulation to sustain cellular redox homeostasis and enhance rice tolerance to Cd toxicity. Application of the NO donor sodium nitroprusside (SNP) weakened JA’s protective effect, establishing NO as a key downstream player in the JA-mediated Cd tolerance pathway [42]. In *Arabidopsis*, exogenous MeJA reduced NO levels in Cd-stressed root tips, and analogous observations have been documented in tomato, indicating the conservation of the JA-NO regulatory module [28,36]. In contrast to JA, SA and NO act cooperatively, and their combined application elicits stronger physiological and biochemical amelioration of Cd toxicity than either compound alone [141]. Evidence further suggests that NO serves downstream of SA signaling, and promotes pectin synthesis, pectin demethylesterification, and lignin biosynthesis, thereby reducing Cd accumulation in rice seedlings [108]. Given the antagonistic relationship between JA and SA, NO may function as a balancing switch mediating their crosstalk [42,108,113] (Figure 3a).

### 4.2. Synergistic Interactions and Multi-Hormonal Networks

Despite their well-documented antagonism, accumulating evidence indicates that JA and SA can act synergistically to mitigate Cd-induced toxicity in plants. In *Bacopa monnieri*, the combined application of SA and JA conferred the greatest alleviation of Cd-induced growth inhibition, pigment loss and oxidative damage, while significantly elevating accumulation of the neuroprotective metabolite bacoside A [142]. In pak-choi grown in Se-rich, high-Cd soil, exogenous co-application of SA and JA synergistically promoted Se biofortification via enhanced uptake, while simultaneously reducing Cd accumulation; this dual modulation outperformed single hormone treatment in optimizing metal chelation capacity and biomass production [53]. Transcriptomic and physiological profiling in the Cd-hyperaccumulator *S. alfredii* further revealed that JA and SA co-application exerts additive protective effects through functionally complementary mechanisms: JA modulates metal transport and chelation, whereas SA activates ROS-scavenging and GSH metabolism [104,143]. This synergy even extends to the rhizosphere microenvironment, where co-application of SA and JA enriches beneficial microbial communities (e.g., Patescibacteria and *Umbelopsis*), establishing a soil–plant–microbe network that improves phytoextraction efficiency and systemic stress tolerance [143].

The JA-SA synergistic alleviation of Cd stress is intricately integrated with multi-hormonal regulation including BRs, auxin (IAA) and abscisic acid (ABA). BRs enhance Cd tolerance through a mechanism that depends on the synergistic activation of both JA and SA pathways. In rice, BR-mediated reduction in Cd accumulation and ROS relies on JA signaling, as evidenced by the loss of BR-mediated protection in the JA-deficient *osmyc2* mutant [144]. Similarly, BR-induced tolerance is diminished in SA-deficient rice lines overexpressing SA hydroxylase genes (*OsS5H1* and *OsS5H2*) [145]. JA and SA also work together to modulate IAA homeostasis to reconcile growth–defense trade-offs. For instance, JA likely restrains root system architecture by antagonizing the IAA pathway after Cd exposure, serving as a protective strategy to reduce metal uptake [146]. SA alleviates Cd stress responses by inhibiting Cd-induced, IAA-mediated ROS production in barley root tips through a comparable regulatory pathway [90]. Abscisic acid (ABA) is a crucial phytohormone involved in plant responses to Cd stress [147], and JA-SA synergy is tightly coordinated with ABA signaling dynamics. In tobacco, Cd tolerance conferred by glandular trichomes involves JAZ10-mediated co-integration of JA and ABA signaling pathways [148]. In mung bean, SA priming increases ABA content to enhance Cd tolerance [126]. In rice, co-application of prohydrojasmonate (PDJ) and silicon (Si) coordinately activates JA and SA pathways while stabilizing ABA biosynthesis and homeostasis to achieve Cd detoxification [149]. As central regulatory nodes, JA and SA integrate these diverse hormonal signals to fine-tune plant responses, maintaining a dynamic equilibrium between stress adaptation and growth under Cd stress (Figure 3b).

## 5. Implications for Phytoremediation and Food Safety

### 5.1. Applications on Phytoremediation

**Figure 4:**
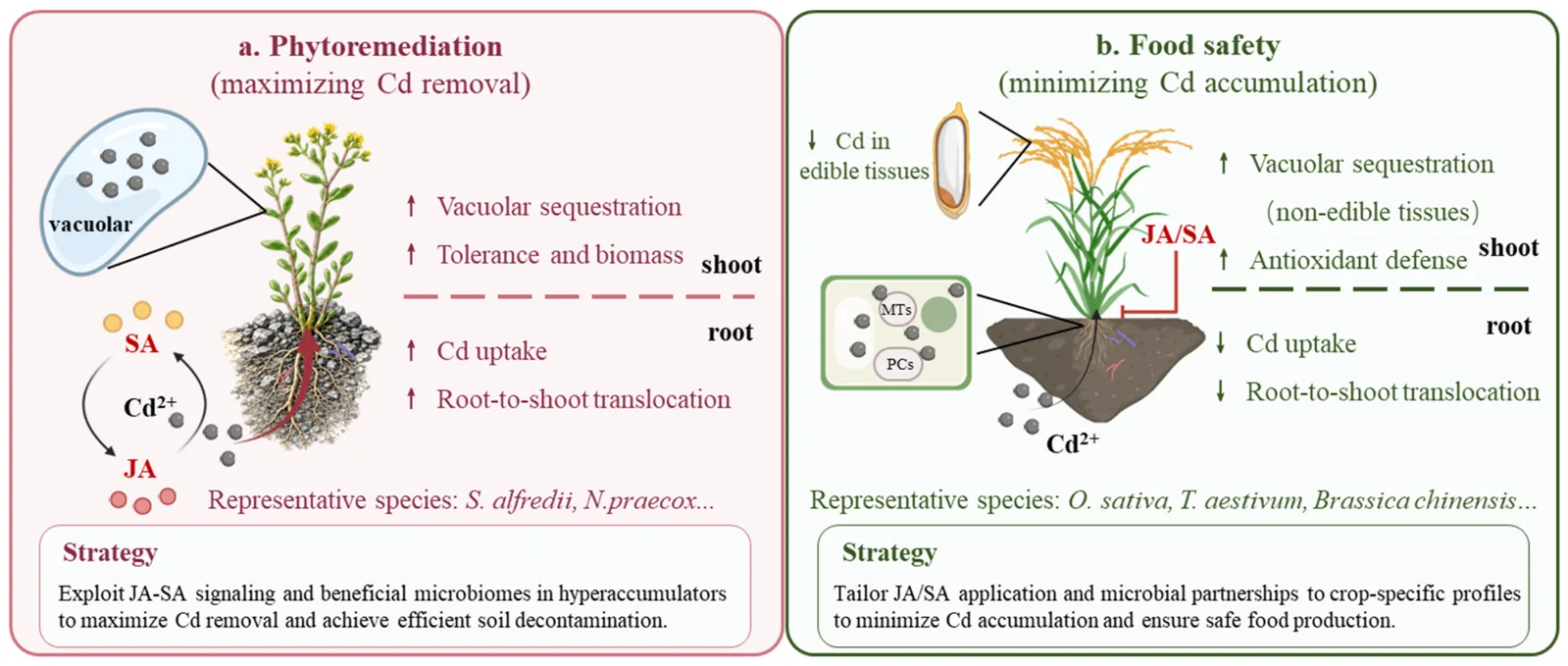
Differential application strategies of JA and SA are categorized based on agricultural and environmental goals. (**a**) Phytoremediation: In hyperaccumulators (e.g., *S. alfredii*, *N. praecox*), JA and SA signaling is exploited to maximize Cd removal from contaminated soils. This strategy involves promoting Cd uptake, enhancing root-to-shoot translocation, and increasing vacuolar sequestration in shoots, modulating rhizosphere microbial communities, coupled with improved biomass and Cd tolerance. (**b**) Food safety: In major crops (e.g., *O. sativa*, *T. aestivum*, *B. chinensis*), the primary objective is to minimize Cd accumulation in edible tissues. Under Cd stress, JA and SA function to suppress Cd uptake and translocation through restricted transport and increased immobilization (e.g., PCs, MTs, cell wall binding), while strengthening the antioxidant defense system and vacuolar sequestration in non-edible tissues to ensure safe food production. ↑ and ↓ means increase and decrease. Arrows and bars indicate promotion and inhibition, respectively.

In phytoremediation systems, the principal objective is to maximize phytoextraction efficiency from contaminated soils. This goal aligns with the inherent physiological traits of Cd hyperaccumulators (e.g., *S. alfredii*, *N. praecox*, and *Arabidopsis halleri*) and certain metal-tolerant non-hyperaccumulating species, which possess elevated capacities for Cd uptake, translocation, and vacuolar sequestration in shoots [140,150,151]. In contrast to conventional crops, these species often exhibit enhanced Cd accumulation and sustained growth in response to exogenous JA and SA treatments [61,103,104,143,152]. For instance, JA and SA have been successfully applied to boost the phytoextraction efficiency of *S. alfredii*, *Nasturtium officinale* and Brassica species [103,153]. Additionally, SA can induce senescence to facilitate the translocation of Cd from roots to shoots in species such as *Festuca arundinacea* and *B. juncea*, thereby enhancing aboveground Cd harvest [153,154,155]. Compared with single exogenous hormone spraying, simultaneous JA and SA treatment in *B*. *monnieri* achieves dual benefits by promoting Cd phytostabilization and stimulating the synthesis of neuroprotective secondary metabolites [142]. Moreover, rhizosphere and endophytic microbes act as critical partners in hormone-mediated phytoremediation. JA and SA application can reshape rhizosphere microbial communities, alter soil pH and enzyme activities, and consequently increase Cd bioavailability for uptake by *S. alfredii* [143]. Endophytic bacteria such as *Stenotrophomonas* SaRB5 and *Pseudomonas fluorescens* enhance Cd phytoextraction in willow and *S. alfredii* by modulating endogenous JA-SA biosynthesis and crosstalk [112]. Thus, exploitation of JA–SA signaling networks within hyperaccumulators and their associated microbiomes offers a robust strategy for efficient soil decontamination (Figure 4a).

### 5.2. Applications on Food Safety

Conversely, in agricultural systems prioritizing food safety, the primary objective is to minimize Cd accumulation in edible plant tissues. This application focuses predominantly on staple and vegetable crops (e.g., rice, wheat, pak-choi, and cauliflower), which typically exhibit contrasting hormonal responses compared to hyperaccumulators. In these crops, exogenous JA and SA generally restrict root Cd uptake, inhibit root-to-shoot translocation, and promote vacuolar sequestration in non-edible tissues [34,39,95,115]. For example, exogenous JA or SA application effectively reduces grain Cd content by regulating key transporters such as OsNramp5, OsHMA3, and OsABCG36 to inhibit absorption and translocation, offering a promising strategy for safe rice cultivation in Cd-polluted soils [34,95]. MeJA or SA treatments in wheat enhance food safety by bolstering antioxidant defenses and optimizing Cd partitioning, producing comparable protective effects [39,115]. In Se-rich, Cd-contaminated soils, co-application of JA and SA in pak-choi not only reduces Cd accumulation but also synergistically enhances Se biofortification [53]. The efficacy of hormone-mediated food safety strategies is significantly potentiated when integrated with beneficial microbes. Co-application of Cd-tolerant Klebsiella strains with exogenous JA alleviates Cd phytotoxicity and impedes Cd translocation from soil to cauliflower roots [156]. Likewise, the endophytic fungus Fusarium *Fo10* relies on the plant SA signaling pathway to modulate Cd uptake, enhance vacuolar sequestration, and promote rhizosphere iron plaque formation, ultimately decreasing Cd levels in rice seeds [157].

It is also noteworthy that species-specific basal hormonal architecture fundamentally governs treatment responsiveness. For instance, *B. juncea* exhibits higher Cd tolerance and accumulation than *B. napus* primarily due to its elevated SA and glutamic acid levels [158]. Conversely, suppression of basal SA signaling paradoxically enhances Cd tolerance in *Arabidopsis* [88]. Therefore, developing precision JA/SA application protocols, which are tailored to the distinct signaling profiles of target crop species and integrated with microbial partners, provides a highly promising avenue for safe crop production in Cd-impacted agroecosystems (Figure 4b).

Despite the well-documented efficacy of exogenous JA and SA in mitigating Cd toxicity under controlled conditions, translating these results to practical agronomic use faces inherent constraints in cost, chemical stability, and environmental variation [159]. Large-scale field application demands repeated spraying at effective concentrations, and the marginal benefits of reduced plant Cd need to exceed the costs of reagents, formulation and labor (Table 1). JA and SA are unstable under field circumstances, prone to photodegradation, oxidation and microbial degradation, and may become inactivated prior to sufficient foliar absorption and systemic signal activation [160,161]. In addition, the efficacy of exogenous JA/SA can be modulated by light, temperature, humidity and soil properties, which may make results obtained from laboratory or single-site trials difficult to reproduce under variable field conditions [162,163].

## 6. Conclusions and Future Perspectives

JA and SA serve as crucial regulatory hubs that orchestrate plant responses to Cd stress. JA enhances plant Cd tolerance primarily by restricting Cd uptake and long-distance translocation, promoting Cd chelation and subcellular compartmentalization, and activating antioxidant defense systems. SA executes analogous protective functions, yet exhibits dual regulatory effects dependent on its concentration, plant species and environmental conditions. Under Cd stress, the JA and SA signaling pathways engage in extensive crosstalk and integrate synergistically with other phytohormone networks, enabling plants to flexibly adjust their defensive strategies in response to the intensity and duration of Cd exposure. Moreover, species-specific responses in JA/SA responsiveness also highlight the importance of trait–context matching in applied breeding and ecological engineering.

Despite advances in understanding JA/SA-mediated Cd tolerance in plants, several key research gaps remain: (i) The precise molecular switches controlling their interaction patterns across diverse Cd concentrations, stress durations and plant species require systematic exploration [164,165]. (ii) The spatial patterns of JA/SA signaling under Cd stress remain unclear. While whole-plant analyses have provided useful information, cell-specific profiling of JA/SA accumulation and signaling in roots and leaves can better clarify their tissue-specific functions [104,166]. (iii) Most exogenous JA/SA data come from controlled seedling experiments, while their efficacy in mature plants under variable field conditions remains poorly understood [16,167]. Multi-factor studies are required to explore how temperature, pH and soil microbes affect JA/SA-dependent Cd tolerance. (iv) Since JA/SA pathways co-regulate plant resistance to pathogens and herbivores, genetic improvements for Cd tolerance may weaken crop biotic stress resistance [165]. These potential trade-offs require comprehensive evaluation.

## Figures and Tables

**Table 1:** Overview of exogenous JA/SA-induced plant protection against Cd stress.

Treatment	Plant Species	Concentration	Corresponding Effects	References
JA	*Oryza sativa*	5 μM	Enhanced Cd tolerance	[25]
		1 mM	Reduced the translocation/accumulation of Cd	[34]
		0.2 μM	Reduced the accumulation of Cd and enhanced Cd tolerance	[40]
		1 μM	Reduced Cd content in the grain	[41,146]
		0.1 μM	Reduced the absorption of Cd	[42]
	*Arabidopsis thaliana*	0.01 μM	Reduced the accumulation of Cd and enhanced Cd tolerance	[28]
	*Malus hupehensis*	5 μM	Reduced the accumulation of Cd	[32]
	*Solanum lycopersicum*	10 μM	Reduced the translocation of Cd	[33]
		2.5; 5 μM	Reduced the accumulation of Cd and enhanced Cd tolerance	[37]
	*Solanum nigrum*	0.01 μM	Reduced the translocation/accumulation of Cd	[35]
	*Cajanus cajan*	100 nM	Enhanced Cd tolerance	[38]
	*Triticum aestivum*	10 μM	Reduced the absorption/translocation of Cd	[39]
		1 μM	Enhanced Cd tolerance	[56]
	*Cosmos bipinnatus*	10 μM	Reduced the absorption of Cd	[43]
		1 μM	Reduced the translocation of Cd	[43,44]
	*Kandelia obovata*	0.1–1 μM	Reduced the absorption/translocation of Cd	[54]
	*Avicennia marina*	1; 10 μM	Reduced the absorption of Cd	[55]
	*Sedum alfredii*	1 μM	Enhanced Cd tolerance and Cd extraction efficiency	[61,143]
	*Brassica napus*	25 µM	Enhanced Cd tolerance	[63]
	*Spinacia oleracea*	25 μM	Reduced the accumulation of Cd and enhanced Cd tolerance	[65]
	*Amaranthus viridis*	5 µM	Reduced the accumulation of Cd and enhanced Cd tolerance	[66]
	*Vicia faba*	0.01 mM	Reduced the accumulation of Cd	[67]
	*Glycine max*	0.01 mM	Enhanced Cd tolerance	[70]
		20 μM	Enhanced Cd tolerance	[71]
	*Ipomoea aquatica*	5 µM	Reduced the accumulation of Cd and enhanced Cd tolerance	[78]
	*Abelmoschus esculentus*	50 μM	Enhanced Cd tolerance	[79]
	*Nicotiana tabacum*	200 μM	Enhanced Cd tolerance	[148]
	*Brassica oleracea*	40 μM	Reduced the absorption of Cd	[156]
JA + Si	*Solanum lycopersicum*	2.5 μM	Reduced the absorption of Cd and enhanced Cd tolerance	[45]
JA + SA	*Brassica chinensis*	50 µM each	Reduced the accumulation of Cd and enhanced Se biofortification	[53]
	*Bacopa monnieri*	75 µM each	Enhanced Cd tolerance and Cd extraction efficiency	[142]
JA + GA	*Cicer arietinum*	1 nM	Reduced the accumulation of Cd and enhanced Cd tolerance	[64]
	*Nicotiana tabacum*	200 μM	Enhanced Cd tolerance	[77]
JA + H_2_S	*Cucumis sativus*	10 μM	Enhanced Cd tolerance	[68]
JA + S	*Brassica juncea*	10 μM	Enhanced Cd tolerance	[69]
JA + SNP	*Triticum aestivum*	10 µM	Reduced the accumulation of Cd and enhanced Cd tolerance	[72]
JA + K	*Pisum sativum*	0.5 mM	Enhanced Cd tolerance	[74]
JA + N	*Mentha arvensis*	1 μM	Enhanced Cd tolerance and nitrogen use efficiency	[75]
JA + Se	*Capsicum annuum*	2.5 μM	Reduced the accumulation of Cd and enhanced Cd tolerance	[76]
SA	*Amaranthus viridis*	100 µM	Reduced the absorption of Cd and enhanced Cd tolerance	[66]
	*Hordeum vulgare*	0.5 mM	Enhanced Cd tolerance	[89]
		0.25 mM	Enhanced Cd tolerance	[90]
	*Triticum aestivum*	0.5 mM	Reduced the absorption of Cd and enhanced Cd tolerance	[91,92,115]
	*Linum usitatissimum*	250; 1000 μM	Reduced the absorption of Cd and enhanced Cd tolerance	[93,117,132]
	*Oryza sativa*	100 μM	Reduced the accumulation of Cd and enhanced Cd tolerance	[95,99,100,145]
		10 μM	Enhanced Cd tolerance	[97,114]
	*Lactuca sativa*	100–200 mg/L	Reduced Cd content in the shoots and enhanced Cd tolerance	[101]
	*Spinacia oleracea*	0.8; 1.6 mM	Reduced Cd content in the shoots and enhanced Cd tolerance	[102]
	*Sedum alfredii*	100 μM	Enhanced Cd tolerance and Cd extraction efficiency	[104,143]
	*Fagopyrum tataricum*	100 μM	Reduced Cd content in the stem and leaf	[110]
	*Solanum lycopersicum*	100 μM	Reduced the accumulation of Cd and changed Cd distribution	[111]
	*Solanum tuberosum*	600 μM	Enhanced Cd tolerance	[116]
	*Cucumis melo*	0.1 mM	Enhanced Cd tolerance	[118]
	*Pterocarya fraxinifolia*	0.5 mM	Reduced the accumulation of Cd and enhanced Cd tolerance	[121]
	*Medicago sativa*	10 μM	Reduced the accumulation of Cd and enhanced Cd tolerance	[124]
	*Vigna radiata*	50 μM	Enhanced Cd tolerance	[125]
		1 mM	Enhanced Cd tolerance	[126]
	*Glycine max*	120 mM	Reduced the absorption/translocation of Cd	[127]
	*Pisum sativum*	500 μM	Enhanced Cd tolerance	[131]
	*Zea mays*	500 μM	Enhanced Cd tolerance	[133,134]
	*Nasturtium officinale*	150 mg/L	Enhanced Cd tolerance and Cd extraction efficiency	[154]
	*Festuca arundinacea*	70 mg/L	Enhanced Cd tolerance and Cd extraction efficiency	[155]
	*Cornus alba*	100 μM	Reduced the translocation of Cd and enhanced Cd tolerance	[161]
SA + SiO_2_NPs	*Oryza sativa*	10 mg/L	Reduced the accumulation of Cd and enhanced Cd tolerance	[98]
SA + SNP	*Oryza sativa*	0.01 μM	Reduced the absorption/translocation of Cd	[109]
		100 μM	Reduced the accumulation of Cd	[108]
		200 µM	Reduced the absorption/accumulation of Cd	[113]
SA + H_2_S	*Brassica juncea*	0.5 mM	Enhanced Cd tolerance	[119]
SA + K	*Chenopodium quinoa*	0.1 mM	Reduced the accumulation of Cd	[120]
SA + EBL + Ca	*Solanum lycopersicum*	100 µM	Reduced the accumulation of Cd and enhanced Cd tolerance	[129]
SA + H_2_O_2_	*Silybum marianum*	0.25 µM	Enhanced Cd tolerance	[130]
SA + ABA	*Festuca arundinacea Brassica juncea*	0.5 mM	Enhanced Cd tolerance and Cd extraction efficiency	[153]

## Data Availability

No new data were created or analyzed in this study.
